# Pre-monsoon spatial distribution of available micronutrients and sulphur in surface soils and their management zones in Indian Indo-Gangetic Plain

**DOI:** 10.1371/journal.pone.0234053

**Published:** 2020-06-02

**Authors:** A. K. Shukla, S. K. Behera, V. K. Singh, C. Prakash, A. K. Sachan, S. S. Dhaliwal, P. C. Srivastava, S. P. Pachauri, A. Tripathi, J. Pathak, A. K. Nayak, A. Kumar, R. Tripathi, B. S. Dwivedi, S. P. Datta, M. C. Meena, S. Das, V. Trivedi

**Affiliations:** 1 ICAR- Indian Institute of Soil Science, Bhopal, Madhya Pradesh, India; 2 ICAR- Indian Agricultural Research Institute, New Delhi, India; 3 Chandra Sekhar Azad University of Agriculture & Technology, Kanpur, Uttar Pradesh, India; 4 Punjab Agricultural University, Ludhiana, Punjab, India; 5 Govind Ballabh Pant University of Agriculture and Technology, Pantnagar, Udham Singh Nagar, Uttarakhand, India; 6 Banda University of Agriculture and Technology, Banda, Uttar Pradesh, India; 7 ICAR-National Rice Research Institute, Cuttack, Odisha, India; 8 ICAR-Indian Agricultural Statistics Research Institute, New Delhi, India; 9 International Zinc Association, New Delhi, India; Ohio State University South Centers, UNITED STATES

## Abstract

The efficient (site-specific) management of soil nutrients is possible by understanding the spatial variability in distribution of phyto-available nutrients (here after called available nutrients) and identifying the soil management zones (MZs) of agricultural landscapes. There is need for delineating soil MZs of agricultural landscapes of the world for efficient management of soil nutrients in order to obtain sustainability in crop yield. The present study was, therefore, undertaken to understand the spatial distribution pattern of available micronutrients (zinc (Zn), boron (B), iron (Fe), manganese (Mn) and copper (Cu)), available sulphur (S), and soil properties (soil acidity (pH), electrical conductivity (EC) and organic carbon (SOC) content) in soils of intensively cultivated Indo-Gangetic Plain (IGP) of India and to delineate soil MZs for efficient management of soil nutrients. Totally, 55101 soil samples from 0–15 cm depth were obtained from 167 districts of IGP during 2014 to 2017 and were analysed for different soil parameters. Soil pH, EC and SOC content varied from 4.44 to 9.80, 0.02 to 2.13 dS m^-1^ and 0.10 to 1.99%, respectively. The concentration of available Zn, B, Fe, Mn, Cu and S varied from 0.01 to 3.27, 0.01 to 3.51, 0.19 to 55.7, 0.05 to 49.0, 0.01 to 5.29 and 1.01 to 108 mg kg^-1^, respectively. Geostatistical analysis resulted in varied distribution pattern of studied soil parameters with moderate to strong spatial dependence. The extent (% area) of nutrient deficiencies in IGP followed the order: S > Zn > B > Mn > Cu > Fe. Principal component analysis and fuzzy c*-*means clustering produced six distinctly different soil MZs of IGP for implementation of zone-specific soil nutrient management strategies for attaining sustainability in crop yield. The developed MZ maps could also be utilized for prioritization and rationalization of nutrients supply in IGP of India.

## Introduction

The Indo-Gangetic Plain (IGP) of India, one of the intensively-cultivated agricultural landscapes of the world, plays a pivotal role in food production of the country. It occupies 52.01 m ha of land area and produces nearly 50% of total food grain production of the country [1]. It is characterized by availability of deep and fertile soils, favourable climatic conditions and sufficient water supply which sustain better agricultural productivity. The IGP played significant role in enhancing food grain production of the country during green revolution era due to growing of nutrient responsive and high yielding crop varieties and adoption of better crop management practices [2]. However, post-green revolution scenario witnessed the decline in factor productivity in IGP, predominantly because of receding ground water table and soil degradation especially due to secondary and micronutrient deficiencies [3]. The emerging deficiency of phyto-available (here after called available) secondary and micronutrients in different parts of the country including IGP is due to less or nil application of organics, over-dependence on straight fertilizers and imbalanced application of nutrients ignoring the replenishment requirement of mined nutrients [4–5].

There are reports of deficiencies of available micronutrients *viz*., zinc (Zn), boron (B), iron (Fe), copper (Cu) and manganese (Mn) [6] and available sulphur (S) [7] in various crops and soils of world. A recent analysis of Indian soils revealed an average deficiency level of 36.5% for Zn, 23.2% for B, 12.8% for Fe, 7.1% for Mn, 4.2% for Cu and 28.5% for S [8]. Availability of micronutrients and S in soils the result of combined influence of native soil nutrient status and important soil properties (pH, electrical conductivity (EC), and soil organic carbon (SOC)). The reduction in crop yield owing to deficit concentration of available micronutrients and S and crop responses to micronutrients and S application in soils of IGP of India have been reported [9–10].

Imbalanced addition of micronutrients and S to soil without the knowledge of their spatial distribution leads to unsustainable crop production. Therefore, proper understanding of spatial distribution of these nutrients and associated soil properties is needed for adoption of efficient (site-specific) soil nutrients management options through variable rate application in order to obtain sustainable crop production [11–12]. Several researchers have investigated spatial distribution variability of available nutrients and associated soil properties in different soils of the world [13–18] at field to regional scale using geostatistics [19–20]. The spatial variations of micronutrients and S availability in soils of IGP is expected to be high primarily due to varied soil types, climatic conditions, crops and crop husbandries. The knowledge pertaining to spatial distribution of available S and micronutrients in soils of IGP is limited.

Geostatistics helps in effective evaluation of the spatial distribution variability of soil properties and available nutrients [21]. Geostatistical estimation predicts the values at unsampled location by establishing spatial correlation between sampled and estimated points and by reducing the estimation error and cost of investigation [22]. The effective way to address the spatial distribution variability of available nutrients in soil for site-specific nutrient management is by delineating soil management zones (MZs) of a particular area [23]. The delineation of MZs involves the techniques like principal component analysis (PCA) and fuzzy clustering, especially fuzzy c*-*means algorithm [24]. Soil MZs have been delineated for enhancing rice crop production in eastern part of India [25], for augmenting oil palm productivity in southern India [18], for improved nitrogen management in wheat in Argentina [26], and for enhancing corn productivity in Chile [27]. However, there is lack in information related to the zone-wise nutrients management in IGP of India. Keeping this in view, we conducted the present study (i) to understand the distribution variability of soil pH, EC, SOC, available S, and micronutrients (Zn, B, Fe, Mn, and Cu) and (2) to delineate potential soil MZs of IGP for site-specific S and micronutrient management, using geostatistical tools.

## Materials and methods

### Study area

**Fig 1:**
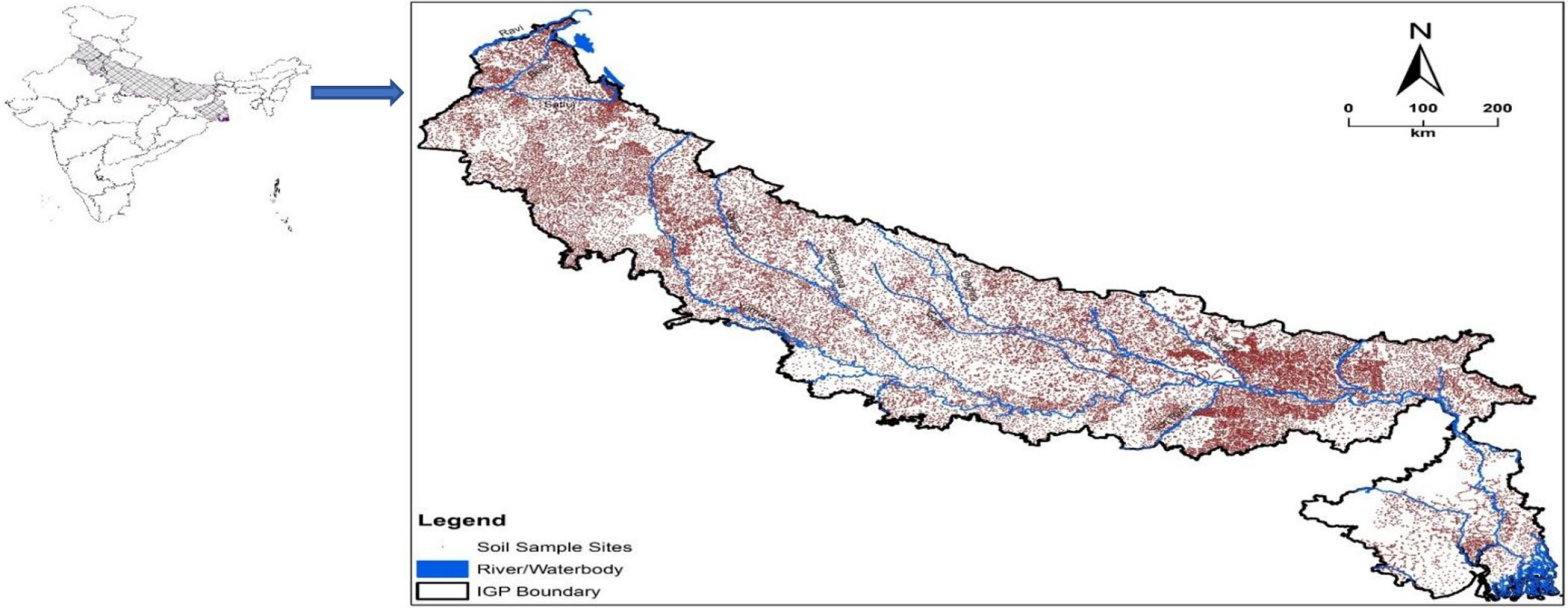
Study location with sampling points.

Indo-Gangetic Plain (located at 21.583° to 32.467° N, 73.833° to 89.817° E) is an extensive fluvial plain spreading in West Bengal, Bihar, Uttar Pradesh, Haryana, and Punjab states of India. The eastern part of IGP is at lower elevation compared to western part. The area is having many rivers which are the sources of alluvium deposited in the plain. For the study purpose, surface soil samples from 0 to 15 cm depth were obtained from the farm lands of IGP (Fig 1). The study area experiences arid (western part), semi-arid (south-western part) and sub-humid (northern, southern and eastern part) climate with average annual rainfall of 300 to 600 mm in arid and semi-arid part and of 600 to 1000 mm in sub-humid part. Majority portion of IGP of India is having hyperthermic temperature regime and experiences mean average temperature of 40°C in summer months and of 10°C in winter months. Soils predominantly fit into Inceptisols, Entisols, Mollisols, Alfisols, and Aridisols orders [28] and having sandy to sandy loam texture [29]. Rice (*Oryza sativa* L.) and wheat (*Triticum aestivum* L.) are the prime crops grown in the area. Other crops include maize (*Zea mays* L.), cotton (*Gossypium* spp.), sugarcane (*Saccharum officinarum* L.), pulses, oil seeds and vegetables.

### Soil sampling and analysis

Under the aegis of “All India Coordinated Research Project on Micro and Secondary Nutrients and Pollutant Elements in Soils and Plants” (AICRP-MSPE), a total of 55101 soil samples were collected by stratified random sampling from both rain-fed and irrigated agricultural soils of small, medium and large land holdings in 167 districts of IGP of India, during April to June months (pre-monsoon period) of 2014 to 2017. Prior to sample collection, necessary permissions were obtained from the owners of the land holdings. It was presumed that there were non-significant changes in available micronutrients and S in soil samples during sampling period. A hand-held stainless-steel auger was used for collection of soil samples. The geographical coordinates (longitude, latitude, and altitude) of each sampling point were recorded using a global positioning system. Composite samples were obtained to reduce the effect of sampling and to enhance the prediction accuracy [30]. Two to 3 subsamples for small holding, 5 to 6 subsamples for medium and 9 to 10 subsamples for large land holdings were collected to make a composite sample. The collected samples were air-dried under shade in a dust-free environment. Stones and debrises were removed before grinding of samples to pass through a sieve of 2 mm size. Processed samples were stored in polythene bottles for analysis.

For conducting analysis of soil samples in laboratory, prior approval was obtained from the Director, ICAR-Indian Institute of Soil Science, Bhopal, India. Soil pH and EC were determined in soil-water suspension (1: 2.5 weight/volume) [31] using pH meter (Make: Eutech, Model: pH 510) and conductivity meter (Make: Hanna, Model: HI 2300), respectively. Estimation of SOC was done by wet oxidation method [32]. Available S concentration was determined by extraction of soil samples with 0.15% calcium chloride (CaCl_2_) [33] and estimation using spectrophotometer (Make: Shimadzu, Model: UV-1800). Available B concentration was estimated through spectrophotometer after extraction with hot water [34]. Available Zn, Fe, Mn and Cu concentration in soil samples were estimated by extracting soils with diethylene-triamine-penta-acetic-acid (DTPA) extractant [35]. Measurement of the micronutrients concentration in the extract was carried out using atomic absorption spectrophotometer (Make: Varian, Model: AA240FS).

### Descriptive statistics

The descriptive statistics viz., mean, minimum, maximum, standard deviation (SD), coefficient of variation (CV), skewness and kurtosis of studied soil parameters were obtained using SAS 9.2 software package [36]. Relationships among the soil parameters were visualized by carrying out Pearson’s correlation analysis.

### Geostatistical analysis

Before geostatistical analysis, the data set was checked for normal distribution by Kolmogorov–Smirnov test. The data set followed the normal distribution. The trend analysis revealed no trend of the data. The analysis for semi-variogram (Eq 1) was obtained using ArcGIS software 10.4.1 for understanding the spatial structure of the soil parameters and to understand the interpolation function [22]. A semi-variogram plots the variance of spatially separated points of data and the separating distance (lag).

$$\gamma (h)=\frac{1}{2m(h)}\overset{m(h)}{\underset{i=1}{\sum }}{\left[Z\left(X_{i}+h\right)-Z\left(X_{i}\right)\right]}^{2}$$ (1)

Where, *γ (h)* is semi-variogram at *h* distance interval; *m(h)* is the sample pair value at *h* distance interval; *Z(X*_*i*_*)*, *Z(X*_*i*_*+h)* are the sample points separated *h* distance. Ordinary kriging (OK) interpolation technique was used for developing distribution maps [37]. In the kriging process, several semi-variogram models were tested for best-fitting. The exponential, stable, K-Bessel and circular models were found best-fitted. These models were selected through cross validation technique which measures the accuracy of the prediction. The root mean square error (RMSE) (Eq 2) which compares the estimated values from semi-variogram and the observed values was used for cross validation.

$$RMSE=\sqrt{\frac{1}{n}\overset{n}{\underset{i=1}{\sum }}{\left[z\left(x_{i},y_{i}\right)-z^{*}\left(x_{i},y_{i}\right)\right]}^{2}}$$ (2)

Where, *z(x*_*i*_, *y*_*i*_*)*, z*(x_*i*_, *y*_*i*_*)* and n denote observed value, predicted value and number of observations, respectively.

Other semi-variogram parameters viz., nugget, sill and range were obtained for available S and micronutrients and associated soil properties. The ratio of nugget: sill is the criterion to describe the nature of spatial dependence [38]. The nugget: sill ratio values of ≤0.25, >0.25 to ≤0.75 and >0.75 denote strong, moderate, and weak nature of spatial dependence, respectively. The distances in which the values of soil parameters are inter-correlated are called ranges.

Principal component analysis was carried out using correlation analysis values as input using SPSS software (version 26.0). Principal components (PCs) with eigenvalues >0.90 were considered for developing the MZs in the present study. A bi-plot using altitude and studied soil parameters was drawn to indicate the effect of altitude on soil nutrients content and to examine relations among altitude and available S and micronutrients. Two to 8 clusters were obtained from the dataset through fuzzy c-means clustering using FUZME software [39]. The membership in each cluster was determined through an iterative process beginning with a random set of cluster means. Each of the observation was provided to the nearest of cluster means. The new mean for each cluster was re-estimated depending upon the distance of the observation from the cluster mean. The distance of the data points to the cluster centre was calculated using the euclidean distance. The optimum cluster number was determined by deriving fuzzy performance index (FPI) (extent of fuzziness) and normalized classification entropy (NCE) (degree of disorganization of specific class) (Eqs 3 and 4). The parameters c, n, μik, log_a_ represents cluster number, observation number, fuzzy membership and natural logarithm, respectively. The differences in the mean values of soil parameters in different MZs were evaluated by variance analysis procedure.

$$\mathrm{NCE}=\frac{n}{n‐c}\left[-\frac{\sum_{k=1}^{n}\sum_{i=1}^{c}\mu_{ik}\log_{a}\left(\mu_{ik}\right)}{n}\right]$$ (3)

$$\mathrm{FPI}=1‐\frac{c}{c‐1}\left[1-\frac{\sum_{i=1}^{c}\sum_{k=1}^{n}{\left(\mu_{ik}\right)}^{2}}{n}\right]$$ (4)

## Result and discussion

### Overall variability of soil properties and available nutrients

**Fig 2:**
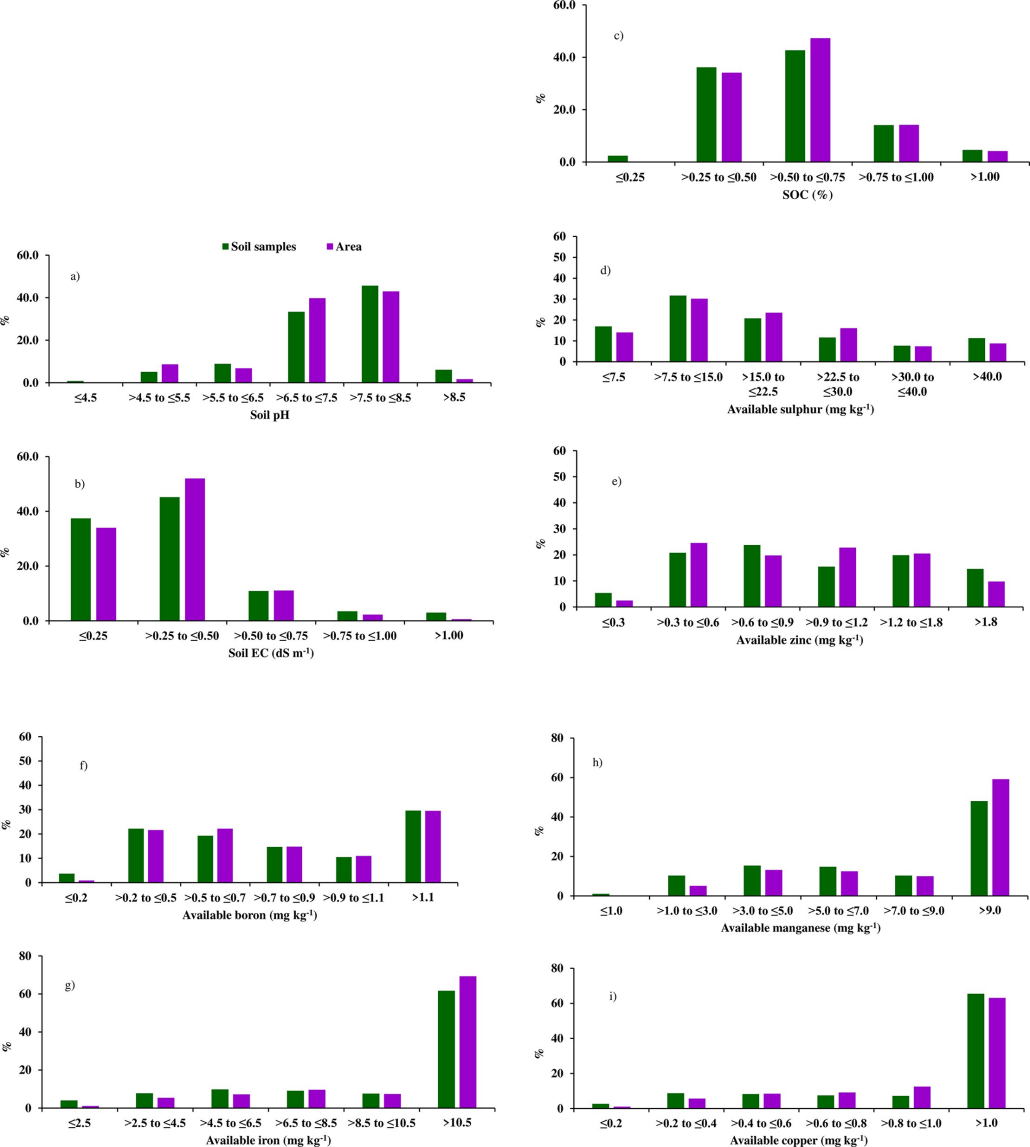
Frequency distribution of soil samples (%) and area (%) under different ranges of available nutrients and associated soil properties of IGP, India.

The IGP of India had acidic (4.04) to alkaline (9.80) soil pH (Table 1) with mean value of 7.41±0.94. About 0.8, 5.1, 8.9, 33.4, 45.7 and 6.1% samples had soil pH ranging ≤4.5, >4.5 to ≤5.5, >5.5 to ≤6.5, >6.5 to ≤7.5, >7.5 to ≤8.5 and >8.5, respectively (Fig 2). The variation in soil pH of the region is attributed to the various soil forming factors especially types of parent materials [40], and prevailing climatic parameters such as mean annual precipitation, temperature and evapotranspiration [41]. The soils of IGP region consisted of both younger and older alluvium [42] and had different units of soil-geomorphic units surrounded by different rivers and landforms. Acid soils were from the parent materials like granite, sandstone and shale. Whereas, limestone parent material gave rise to neutral to basic soils. Moreover, the proportion of mean annual precipitation and evaporation affects soil pH changes. Excess of evaporation compared to precipitation in IGP region resulted in accumulation of Ca^2+^ in soil surface resulting in higher soil pH. Whereas higher precipitation compared to evaporation results in leaching of Ca^2+^ and accumulation of Al^3+^ in surface soil resulting in lower soil pH. Soils were non-saline (EC 0.02 dS m^-1^) to slightly saline (EC 2.13 dSm^-1^) in nature with mean EC value of 0.35±0.26 dS m^-1^. About 37.4 and 45.2% soil samples had EC value of ≤0.25 and >0.25 to ≤0.50 dS m^-1^, respectively. Some parts of IGP had higher salt concentration in soil because of low precipitation, higher evaporation and irrigation of crops with saline water and poor soil-crop management. Soil organic carbon is an integral part of soil organic matter which influences soil physical, biological and chemical properties. The level of SOC in IGP region varied widely from 0.10% to 1.99% with mean value of 0.58±0.24% (Table 1). SOC content in 36.2, 42.7 and 14.1% samples were >0.25 to ≤0.50, >0.50 to ≤0.75 and >0.75 to ≤1.00%, respectively. Most parts of the IGP had low SOC levels because of imbalanced use of fertilizers, nil or lees crop residue addition, and adoption of excessive tillage practices [43].

**Table 1:** Descriptive statistics of available nutrients and associated soil properties of IGP, India (n = 55101).

Soil parameters	Minimum	Maximum	Mean	SD	CV (%)	Skewness	Kurtosis
pH	4.04	9.80	7.41	0.94	12.6	-0.98	1.03
EC (dS m^-1^)	0.02	2.13	0.35	0.26	74.6	1.73	3.31
SOC (%)	0.10	1.99	0.58	0.24	40.8	0.98	1.21
S (mg kg^-1^)	1.01	108	20.8	17.9	85.7	2.01	4.43
Zn (mg kg^-1^)	0.01	3.27	1.06	0.61	57.2	0.72	-0.35
B (mg kg^-1^)	0.01	3.51	0.97	0.68	70.0	1.32	1.21
Fe (mg kg^-1^)	0.19	55.7	17.9	12.6	70.3	0.69	-0.51
Mn (mg kg^-1^)	0.05	49.0	12.5	10.4	83.0	1.22	0.65

SD = standard deviation, CV = coefficient of variation, pH = soil acidity, EC = electrical conductivity, SOC = soil organic carbon, S = available (calcium chloride extractable) sulphur, Zn = available (DTPA extractable) zinc, B = available (hot water soluble) boron, Fe = available (DTPA extractable) iron, Mn = available (DTPA extractable) manganese, Cu = available (DTPA extractable) copper.

The soils of IGP had mean concentration of 20.85±17.87 mg kg^-1^ for available S (varied from 1.01 to 107.52 mg kg^-1^), 1.06±0.61 mg kg^-1^ for available Zn (varied from 0.01 to 3.27 mg kg^-1^) and 0.97±0.68 mg kg^-1^ for available B (varied from 0.01 to 3.51 mg kg^-1^) (Table 1). The mean concentrations were 17.94±12.61 mg kg^-1^ for available Fe (varied from 0.19 to 55.71 mg kg^-1^), 12.47±10.36 mg kg^-1^ for available Mn (varied from 0.05 to 49.02 mg kg^-1^) and 1.74±1.19 mg kg^-1^ for available Cu (varied from 0.01 to 5.29 mg kg^-1^). The mean concentrations of available nutrients followed the order: available S > available Fe > available Mn > available Cu > available Zn > available B. According to the critical concentration ranges proposed by Shukla and Tiwari [44], the concentration of available S in 16.9% sample of IGP region was ≤7.5 mg kg^-1^, in 31.7% sample >7.5 to ≤15.0 and in 20.8% sample >15.0 to ≤22.5 mg kg^-1^ (Fig 2). The concentration of available micronutrients was as follows: ≤0.6 mg Zn kg^-1^ in 26.2% sample, ≤0.5 mg B kg^-1^ in 25.99% sample, ≤4.5 mg Fe kg^-1^ in 11.8% sample, ≤3.0 mg Mn kg^-1^ in 11.4% sample and ≤0.2 mg Cu kg^-1^ in 2.7% sample. In line with our observations, Singh et al. [45] reported available S concentration of 1.4 to 90.3 mg kg^-1^ in north-western IGP soils and west-Himalayan soils of India. Shukla et al [17] also recorded the concentration of available S ranging 0.55 to 130 mg kg^-1^ in soils of Shiwalik Himalayan region (SHR), India. The concentrations of available Zn, Fe, Mn and Cu were recorded ranging 0.10 to 8.00, 0.12 to 48.8, 0.53 to 26.6 and 0.10 to 7.97 mg kg^-1^ respectively, in soils of Trans-Gangetic Plain (TGP), India [12] and ranging 0.14 to 2.35, 0.90 to 28.5, 0.81 to 24.4 and 0.09 to 2.34 mg kg^-1^ respectively, in the soils of a Deccan Plateau Region (DPR), India [47]. The variations in the concentration of available B (hot water-soluble B) ranging 0.01 to 2.92 mg kg^-1^ in some acid soils [46] and ranging 0.19 to 6.11 mg kg^-1^ in some Indian Alfisols and Vertisols [47] were also reported. This variation in S and micronutrient concentration in IGP region is primarily because of nature of parent material, types of crops grown and amount and type of fertilizers applied in the region. The native supply of S and micronutrients is determined by weathering of the parent material [48] The organic acids released from the decomposition of crop residue as well as by the microbes facilitate the weathering of soil minerals and thus nutrient release. Several researchers have also reported variations in S and micronutrient concentration in soils of IGP due to diversity in crops grown and adoption of different of soil-crop management practices in the region [49–51]. The CV values of studied soil parameters varied from 12.64% (pH) to 85.72% (available S) revealing their moderate variability [52]. This is in parallel to the observations of Bogunovic et al. [16] who recorded low, moderate, and high variability for pH, EC and SOC, respectively, in Rasa river valley soils of Croatia. Similarly, low variability for soil pH, and moderate variability for SOC and available Fe were reported by Tesfahunegn et al. [13] in northern Ethiopian soils. Wang et al. [53] recorded moderate variability for available Zn, Fe, Cu, and Mn in paddy soils of China.

### Relationship among soil properties and available nutrients

There were significant correlations among the studied soil parameters (Table 2) although some correlations had low correlation coefficient values. Lower but significant correlation coefficient values are because of larger sample population. There was positive correlation (p ≤ 0.01) of soil pH with EC, available S, and B and negative correlation (p ≤ 0.01) with SOC, available Zn, Fe, Mn and Cu (Table 2) in the IGP region. Soil pH is the key variable influencing soil chemical, biological and physical properties [54]. It modifies soil chemical processes and thereby nutrient forms and their phyto-availability. The formation of free metallic cations and protonated anions is favoured by low pH condition [55, 56]. Whereas higher pH supports carbonate and hydroxyl complexes formation. Hence, the availability of cations increases with increase in soil acidity and availability of anions decreases with decrease in soil pH. The negative correlation of soil pH with available Zn, Fe, Mn and Cu explained the reduction in availability of these nutrients in IGP region with increase in soil pH. The negative relationship of soil pH with available cationic micronutrients was recorded by Wei et al., [57] in loess plateau soils of China and by Katyal et al. [58] and Shukla et al., [12] in some Indian soils. Soil EC was positively correlated (p ≤ 0.01) with all the studied soil parameters except SOC and available Mn. Corwin & Lesch, [59] reported EC as an indicator of phyto-available nutrients and soil salinity. The SOC content was negatively correlated (p ≤ 0.01) with available S and B and positively correlated (p ≤ 0.01) with available Zn, Fe, Mn and Cu (Table 2). The negative correlation of SOC with available S is in contradiction with the observation that soil organic fractions often serve as an important source of plant available S in soil [60, 61]. However, availability of S in soil depends upon the mineralization and immobilization process which in turn influenced by types of soil, crop managements and microbial activities [62]. The positive correlation of SOC with available cationic micronutrients indicates higher availability of these nutrients with increase in SOC content. Soil organic matter (in which SOC is a main component) releases chelated which enhances phyto-availability of these nutrients. Available S was positively correlated (p ≤ 0.01) with available Zn and B and negatively correlated (p ≤ 0.01) with available Fe, Mn and Cu. The correlations of available Zn with available B, Fe, Mn and Cu (p ≤ 0.01) were positive. There was negative correlation of available B with available Fe (p ≤ 0.01), Mn (p ≤ 0.05) and Cu (p ≤ 0.01). Available Fe was positively correlated (p ≤ 0.01) with available Mn and Cu. There was positive correlation of available Mn with available Cu (p ≤ 0.01). The positive correlations among the cationic micronutrients indicate that similar sets of factors influence distribution of these nutrients in IGP region. Behera and Shukla [63] also recorded positive correlations among the phyto-available cationic micronutrients in Indian acid soils.

**Table 2:** Correlation (Pearson’s) coefficients revealing relationship among available nutrients and associated soil properties of IGP, India (n = 55101).

Soil parameters	pH	EC	SOC	S	Zn	B	Fe	Mn	Cu
pH	1.000	** **	** **		** **	** **	** **	** **	** **
EC	0.053**	1.000							
SOC	-0.120**	-0.028**	1.000						
S	0.122**	0.102**	-0.057**	1.000					
Zn	-0.017**	0.082**	0.014**	0.134**	1.000				
B	0.089**	0.079**	-0.060**	0.210**	0.101**	1.000			
Fe	-0.303**	0.025**	0.139**	-0.073**	0.135**	-0.127**	1.000		
Mn	-0.291**	-0.027**	0.120**	-0.046**	0.106**	-0.010*	0.510**	1.000	
Cu	-0.169**	0.012**	0.194**	-0.043**	0.157**	-0.111**	0.496**	0.414**	1.000

pH = soil acidity, EC = electrical conductivity, SOC = soil organic carbon, S = available (calcium chloride extractable) sulphur, Zn = available (DTPA extractable) zinc, B = available (hot water soluble) boron, Fe = available (DTPA extractable) iron, Mn = available (DTPA extractable) manganese, Cu = available (DTPA extractable) copper

* and ** indicate significant at p ≤ 0.05 and p ≤ 0.01, respectively.

### Spatial structure of soil properties and available nutrients

**Fig 3:**
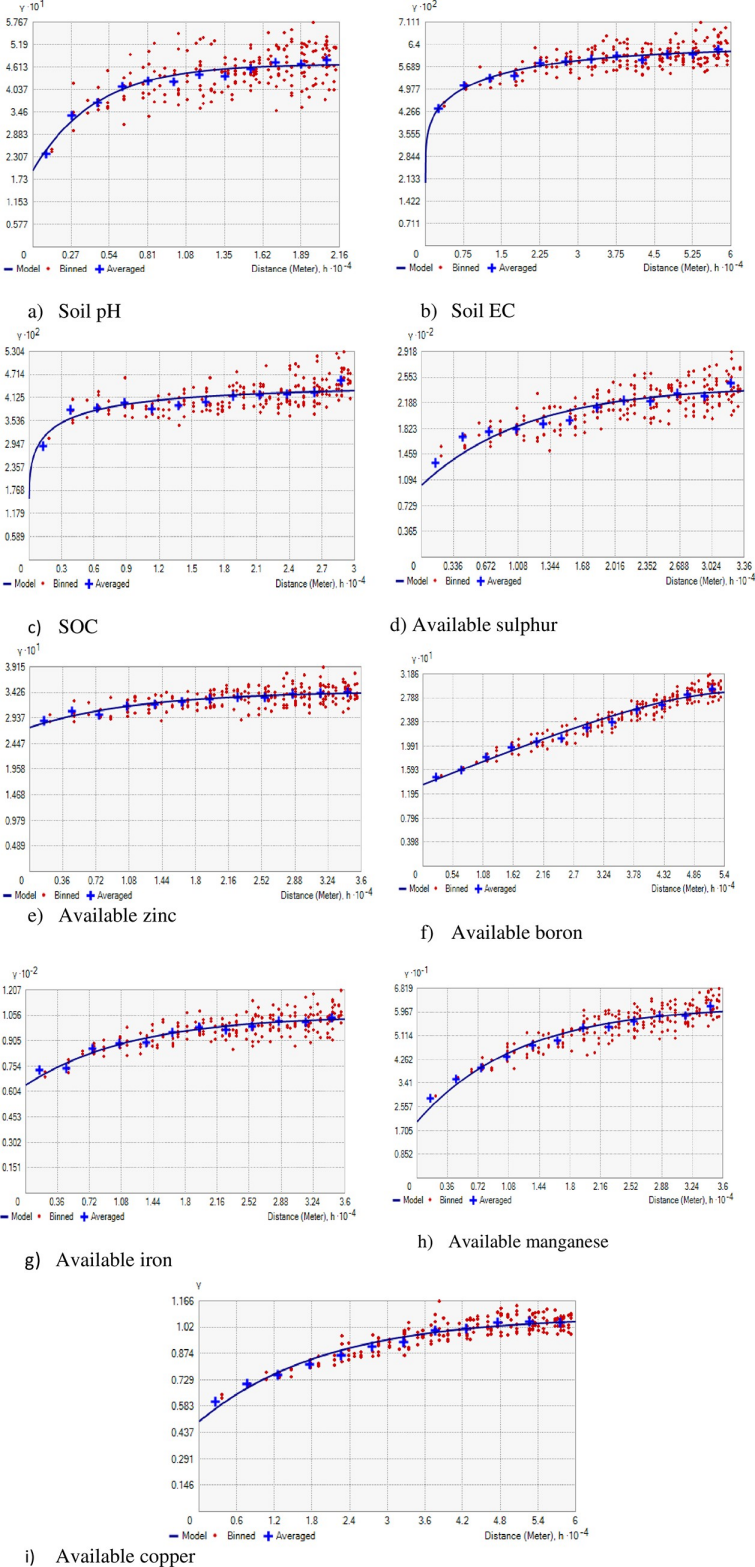
Semi-variograms of available nutrients and associated soil properties of IGP, India.

Geostatistical analysis revealed the best fitted exponential model for soil pH, available Fe, Mn and Cu, stable model for EC, SOC and available Zn, K-Bessel for available S and circular for available B with lower RMSE values (Table 3, Fig 3). The nugget value, indicating micro-variability, was higher for available S, Fe and Mn. This is attributed to inability of the sampling distance to capture spatial dependence. Nugget: sill ratio varied from 0.25 (SOC) to 0.61 (available Fe) with strong (SOC) to moderate (rest of soil parameters) spatial dependence (Table 3). The strong spatial dependence is because of intrinsic characters like soil mineralogy. On the other hand, moderate spatial dependence is due to combined influence of both intrinsic soil characters and extrinsic factors like effect of crops grown and fertilization.

**Table 3:** Parameters of semi-variogram models for available nutrients and associated soil properties of IGP, India.

Soil parameter	Model	Nugget	Partial sill	Sill	Nugget/Sill	Spatial dependence	Range (m)	RMSE
pH	Exponential	0.18	0.28	0.46	0.39	Moderate	13115	1.03
EC (dS m^-1^)	Stable	0.02	0.04	0.06	0.33	Moderate	36000	1.02
SOC (%)	Stable	0.01	0.03	0.04	0.25	Strong	30000	1.03
S (mg kg^-1^)	K-Bessel	102	140	242	0.42	Moderate	33109	1.17
Zn (mg kg^-1^)	Stable	0.21	0.14	0.35	0.60	Moderate	48000	0.92
B (mg kg^-1^)	Circular	0.13	0.16	0.29	0.45	Moderate	54000	1.08
Fe (mg kg^-1^)	Exponential	64.14	41.43	105.5	0.61	Moderate	36000	0.89
Mn (mg kg^-1^)	Exponential	19.6	42.5	62.1	0.32	Moderate	36000	1.13
Cu (mg kg^-1^)	Exponential	0.47	0.62	1.09	0.43	Moderate	60000	0.91

pH = soil acidity, EC = electrical conductivity, SOC = soil organic carbon, S = available (calcium chloride extractable) sulphur, Zn = available (DTPA extractable) zinc, B = available (hot water soluble) boron, Fe = available (DTPA extractable) iron, Mn = available (DTPA extractable) manganese, Cu = available (DTPA extractable) copper, RMSE = Root mean square error.

The range value indicates the distance within which the samples are related spatially. The range values were 13115 m for pH, 36000 m for EC, available Fe and Mn, 30000 m for SOC, 33109 m for available S, 48000 m for available Zn, 54000 m for available B and 60000 m for available Cu. Soil parameters with higher range values are affected by natural as well as anthropogenic factors to a greater distance than the soil parameter with lower range value [64]. The variations in range values of pH, EC, SOC, available S, Zn, B, Fe, Mn and Cu is attributed to joint action of parent material, climatic condition and various land management practices. In line with the current study, other authors recorded ranges values of 132,000 m for pH, 65,000 m for EC, 59,000 m for SOC, 82,000 m for available S, 66 000 m for available Zn and 82,000 m for available Fe in SHR [17] and of 32, 490 m for available Zn, 61, 400 m for available Cu, 5, 370 m for available Mn and 140, 000 m for available Fe in intensively cultivated TGP, India [12]. The range values of the studied soil parameters in this study could be used as guide for designing future sampling strategies in similar regions. Though the ideal sampling interval needs to be less than half of semi-variogram range [30], it is hereby recommended to have shorter sampling distance than the range value obtained in this investigation for future studies characterizing spatial variability of pH, EC, SOC, available S, Zn, B, Fe, Mn and Cu in similar regions.

**Fig 4:**
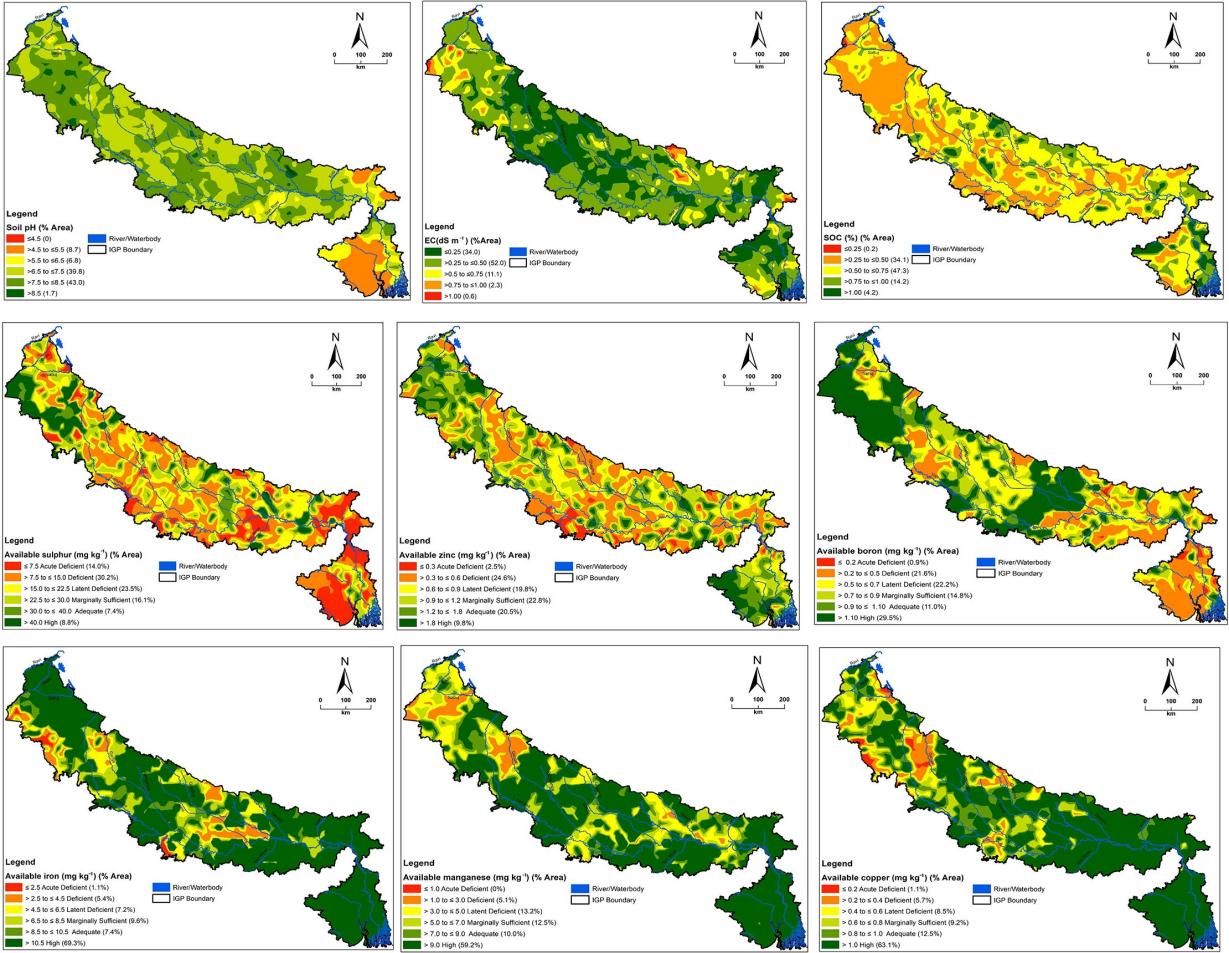
Spatial distribution maps of available nutrients and associated soil properties of IGP, India.

The distribution maps (generated by OK) displayed varied distribution patterns of soil properties, available S and micronutrients in IGP region of India (Fig 4). About 8.7, 6.8, 39.8 and 43.0% area were having soil pH ranging >4.5 to ≤5.5, >5.5 to ≤6.5, >6.5 to ≤7.5 and >7.5 to ≤8.5, respectively. The area having soil pH >4.5 to ≤6.5 is predominantly found in eastern part of IGP. Soils with pH values between >6.5 to ≤7.5 is optimum for plant growth and development. However, the areas having soil pH ≤6.5 (15.5%) and >7.5 (44.7%) need soil management practices for better crop production. There is need to adopt befitting liming technologies along with suitable crop cultivars in acid soils areas. Majority portion of IGP had EC ranging ≤0.25 (34.0% area) and >0.25 to ≤0.50 (52.0% area) dS m^-1^. Soil salinity was recorded in some pockets of IGP. However, soil sodicity (viz. presence of higher percentage of exchangeable sodium) is prevalent in northern and north-western part of IGP. The soil forming process of clay illuviation leads to development of soil sodicity [65]. The repeated cycles of drying and wetting of soils cause hydrolysis of feldspar causing release of alkalis. This results in calcium carbonate precipitation at high soil pH and generation of sub-soil acidity. Proper soil-crop management practices such as irrigation with good quality water, addition of soil amendments, and growing of salt tolerant crop cultivars with matching crop-nutrient management options need to be adopted in IGP areas having higher soil pH (> 7.5) [66]. The status of SOC was low (≤0.50%) in 34.3%, medium (>0.5 to ≤0.75%) in 47.3% and high (>0.75%) in 18.4% area of IGP. A large portion of area in western and southern IGP had low SOC status. The status of SOC was medium in most area of northern, central and eastern IGP. The variations in SOC status in different parts of IGP is the combined influence of soil types, climatic conditions, types of crops grown and adoption of different soil-crop management practices. Bhattacharyya et al. [67] recorded variations in SOC status in different parts of IGP. Efforts must be done to enhance SOC content in the areas of IGP having low and medium SOC status. It could be achieved by addition of organics, adoption of crop diversification practices by including pulse crops in cereal based cropping systems [68] and by following conservation agriculture practices [69].

Available S was acutely deficient in 14.0% area, deficient in 30.2% area and latently deficient in 23.5% area. More area in eastern IGP had S deficiency. The variations in available S concentrations in different parts of IGP is because of differences in inherent S content in soils, soil properties, climatic conditions, soil-crop management practices and mismatch between S addition and S removal under various crops and/or cropping systems [70]. The management of soil S needs to be prioritized in the areas having S deficiency. About 2.5, 24.6 and 19.8% area had available Zn concentration ranging ≤0.3 (acute deficient), >0.3 to ≤0.6 (deficient) and >0.6 to ≤0.9 mg kg^-1^ (latent deficient), respectively. The deficiency Zn was prevalent in almost all parts of IGP except eastern and western parts. The concentration of available B was deficient (including acute deficient) (≤0.5 mg kg^-1^) in 22.5% area. About 22.2% area was latently deficient (>0.5 to ≤0.7 mg kg^-1^)) in available B. Boron deficiency was prevalent in eastern and some pockets of northern, southern and western part of IGP. Available Fe was deficient in 6.5% area and latently deficient in 7.2% area of IGP. Some pockets of southern and central part of IGP had Fe deficiency. About 5.1 and 13.2% area had available Mn concentration ranging >1.0 to ≤3.0 (deficient) and >3.0 to ≤5.0 mg kg^-1^ (latent deficient), respectively. The deficiency of Mn was found in some parts of western IGP. The concentration of available Cu was ≤0.2 mg kg^-1^ (acute deficient) in 1.1% area, >0.2 to ≤0.4 mg kg^-1^ (deficient) in 5.7% area and >0.4 to ≤0.6 mg kg^-1^ (latent deficient) in 8.5% area. Some pockets of western, northern and southern IGP had Cu deficiency. The spatial diversity in micronutrients concentration in different parts of IGP is because of physiographic variations. Moreover, cultivation of different crops, adoption of different soil-crop management practices and non-addition of micronutrient fertilizers add to the different spatial distribution scenario of micronutrients. The crops responses to S and micronutrient application varied to a greater extent in different parts of IGP (Table 4) [71]. This is due to varied concentration of phytoavailable S and micronutrients, nature crop grown and amount of nutrients applied. For example, the response of cereal crops ranged from 0.05 to 1.06 t ha^-1^ for S, 0.01 to 5.47 t ha^-1^ for Zn, 0.01 to 4.40 t ha^-1^ for Fe, 0.01 to 3.78 t ha^-1^ for Mn, 0.01 to 1.78 t ha^-1^ for Cu and 0.01 to 1.67 t ha^-1^ for B. However, the response rates of other group of crops were different. This warrants site-specific S and micronutrients application based on their phyto-availability and crop requirement for sustainable crop production. Therefore, the developed spatial distribution maps could be used by the farmers, farm managers, policy makers, fertilizer industries, planners and extension agencies for understanding the deficiencies of S and micronutrients in different parts of IGP. Accordingly, right kind and quantity of S and micronutrients fertilizer could be produced and distributed for their rational and site-specific application.

**Table 4:** Crop responses to S and micronutrient application in different states in IGP, India.

Nutrient	State	Crop	No. of trials	Response range (t ha^-1^)
S	Punjab	Cereals	6	0.05 to 1.06
		Oilseed (Groundnut)	3	0.07 to 0.31
	West Bengal	Oilseed (Rapeseed)	2	0.01 to 0.48
Zn	Bihar	Cereals	1004	0.01 to 3.43
		Millets	7	0.03 to 0.92
		Pulses	13	0.03 to 0.87
		Oilseed (Groundnut)	2	0.31 to 0.63
		Vegetable (Onion)	2	4.35 to 8.70
		Cash crop (Sugarcane)	1	19.10
	West Bengal	Cereals	15	0.02 to 4.79
	Haryana	Cereals	557	0.02 to 3.21
		Millets	2	0.01 to 0.67
		Oilseed (Groundnut)	1	0.21
		Cash crop (Cotton)	2	0.06 to 0.34
	Punjab	Cereals	952	0.01 to 5.47
		Millets	30	0.01 to 0.50
		Pulses	29	0.05 to 0.69
		Oilseeds	27	0.04 to 0.42
		Vegetables	2	0.01 to 3.16
		Cash crops	28	0.01 to 24.6
	Uttar Pradesh	Cereals	177	0.01 to 1.26
Fe	Bihar	Cereals	17	0.01 to 1.20
		Pulses	7	0.01 to 0.80
		Millets	2	0.25 to 0.77
		Vegetables	2	0.50 to 1.53
	Haryana	Cereals	2	0.16 to 1.10
	Punjab	Cereals	10	0.54 to 4.40
		Millets	5	0.03 to 0.31
		Pulses	2	0.07 to 0.82
		Cash crops	2	6.20 to 7.20
Mn	Bihar	Cereals	143	0.01 to 1.78
		Vegetables	2	3.63 to 4.30
		Cash crop (Sugarcane)	1	1.78
	Haryana	Cereal (Wheat)	5	0.12 to 0.24
	Punjab	Cereal (Wheat)	5	0.20 to 3.78
Cu	Bihar	Cereals	144	0.01 to 1.78
		Vegetable (Onion)	2	4.43 to 6.18
	Haryana	Cereals	5	0.01 to 0.20
B	Bihar	Cereals	144	0.01 to 1.67
		Pulses	8	0.03 to 0.90
		Oilseed (Groundnut)		
		Vegetables	3	0.67 to 5.80
		Cash crop (Sugarcane)	1	1.81
	West Bengal	Vegetables	25	0.50 to 6.24
	Punjab	Oilseed (Groundnut)	7	0.05 to 0.36
		Cash crop (Cotton)	2	0.06 to 0.35

### Soil management zones

**Fig 5:**
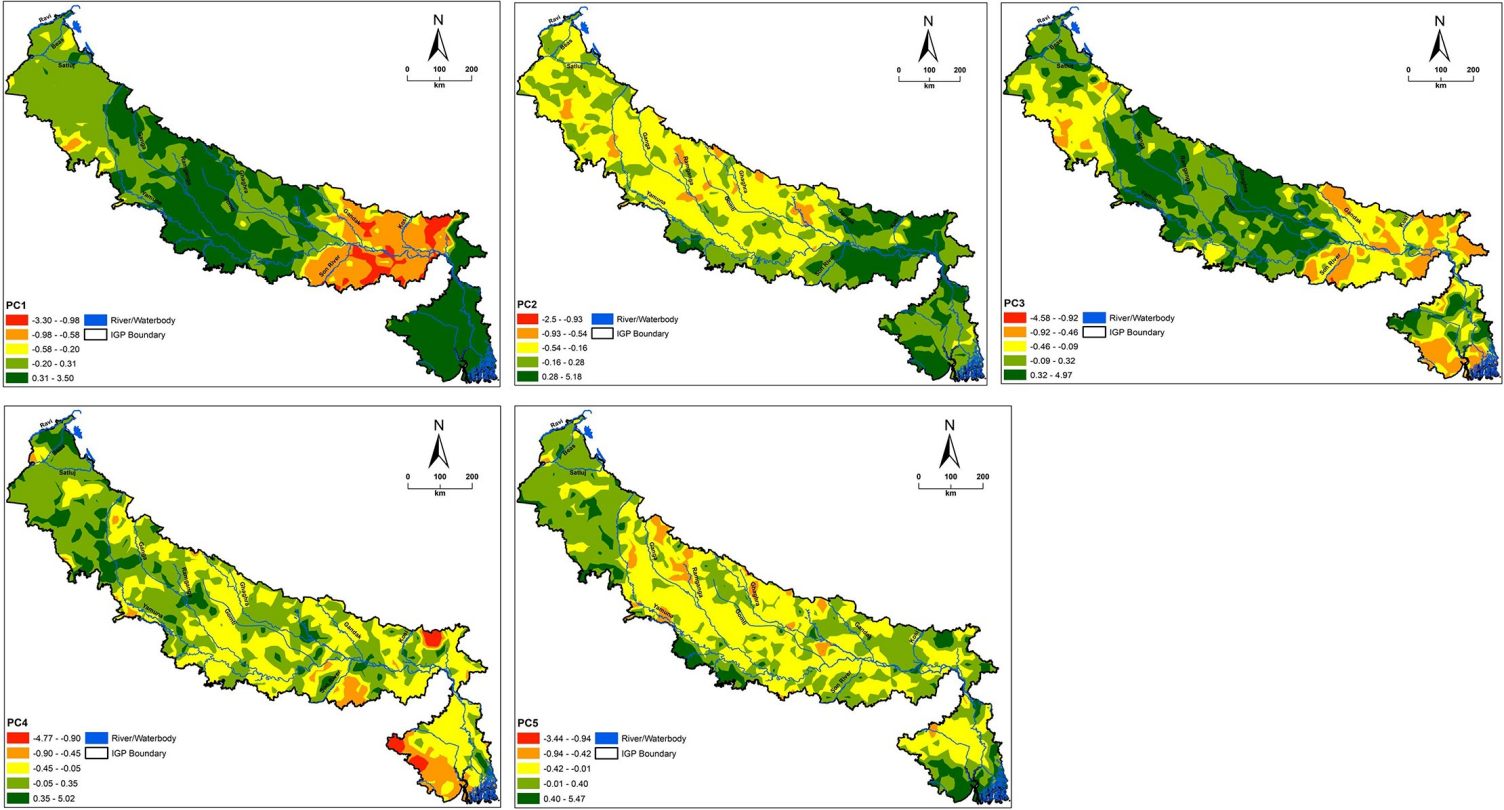
Kriged maps of first five principal components.

**Fig 6:**
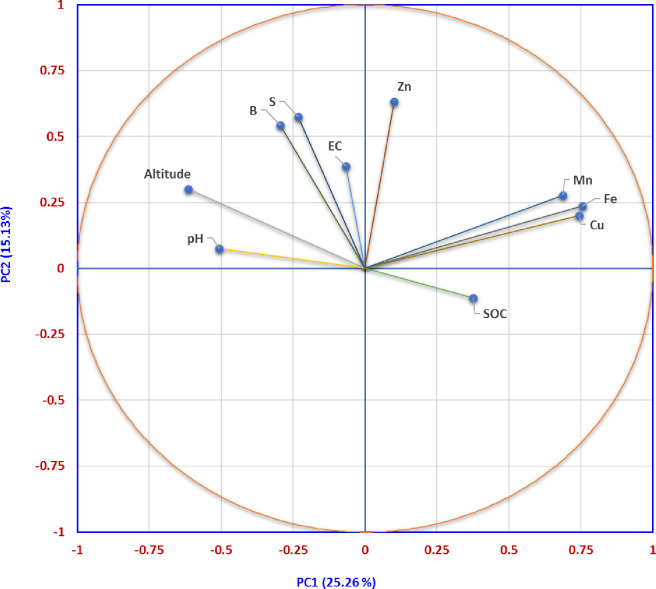
PC1 and PC2 bi-plot of soil parameters and altitude of soil samples.

Principal component analysis, aggregating and summarizing the variability in the nine studied soil parameters and altitude, resulted in ten PCs (Table 5). Out of which, first five PCs having eigenvalue > 0.90 and accounting for 69.62% of total variance were considered (Table 5, Fig 5). The PC1 explained 25.26% of total variance and dominated with pH, available Mn, Fe and Cu and altitude. Additionally, PC2, PC3, PC4 and PC5 explained 15.13, 10.35, 9.86 and 9.01%, respectively, of total variance. Principal component 2 was dominated with available S, Zn and B, PC3 by EC, PC 4 by SOC and PC5 by available Zn. PC1 and PC2 bi-plot of revealed 3 groupings (Fig 6). Soil pH, altitude, EC, available S and B formed one group and available Zn, Fe, Mn and Cu another. Contrastingly, Shukla et al. [17] recorded the grouping of altitude with SOC, available Zn, Mo, Cu, Mn, and Fe in SHR, India. Soil pH, EC, available S and B formed another group. This is mainly attributed to the differences in soil types and prevailing climatic conditions of both the regions.

**Table 5:** Principal component analysis of soil parameters and loading coefficient for the first five PCs.

Principal Component		Eigenvalues				Component Loading (%)				Cumulative Loading (%)		
PC1		2.526				25.263				25.263		
PC2		1.513				15.132				40.395		
PC3		1.035				10.351				50.746		
PC4		0.986				9.862				60.608		
PC5		0.901				9.010				69.618		
PC6		0.837				8.372				77.990		
PC7		0.766				7.663				85.653		
PC8		0.661				6.614				92.267		
PC9		0.451				4.511				96.778		
PC10		0.322				3.222				100.00		
**PC loading for each variable**												
	pH	EC	SOC	S	Zn		Fe	Cu	Mn		B	Altitude
PC1	**-0.507**	-0.067	0.375	-0.232	0.101		**0.756**	**0.743**	**0.687**		-0.294	**-0.614**
PC2	0.075	0.389	-0.112	**0.574**	**0.632**		0.237	0.200	0.278		**0.542**	0.300
PC3	0.450	**0.606**	0.271	0.118	0.019		-0.092	0.211	-0.279		-0.291	-0.344
PC4	0.139	-0.475	**0.707**	0.292	0.026		-0.165	0.050	-0.073		0.269	-0.091
PC5	0.421	-0.465	-0.267	-0.051	**0.513**		0.029	0.179	-0.068		**-0.366**	-0.003

pH = soil acidity, EC = electrical conductivity, SOC = soil organic carbon, S = available (calcium chloride extractable) sulphur, Zn = available (DTPA extractable) zinc, B = available (hot water soluble) boron, Fe = available (DTPA extractable) iron, Mn = available (DTPA extractable) manganese, Cu = available (DTPA extractable) copper.

**Fig 7:**
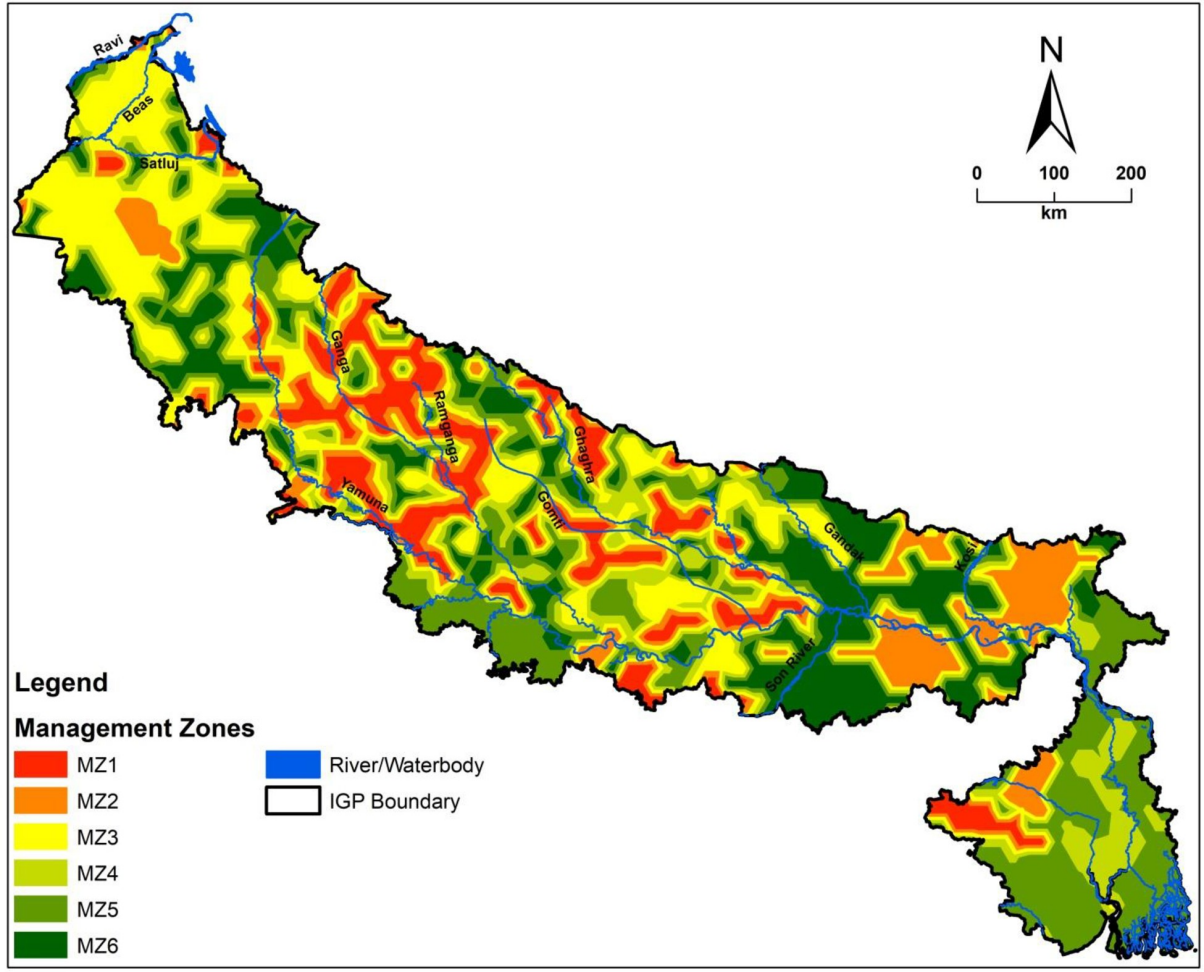
Sulphur and micronutrient management zones of IGP, India.

The clustering was carried out by considering first 5 PCs. This resulted in 6 clusters considering minimum FPI and NCE values (Table 6) and six nutrient MZs (Fig 7). This result is in line with the findings of Fu et al. [72] (in Sanjiang plain of China), Davatgar et al. [73] (in paddy cultivated areas of Iran), Tripathi et al. [25] (in paddy growing soils of India), Shukla et al. [17] (in SHR, India) and Shukla et. al. [46] (in a DPR, India). The % of area in different MZs was in the order: MZ5 (24.4%) > MZ3 (21.7%) > MZ4 (16.2%) > MZ6 (14.8%) > MZ2 (14.1%) >MZ1 (8.8%). The 6 MZs were different form one another and they had different soil properties and available S and micronutrient concentration (Table 7). This is attributed to the variations in soils, agro-ecological conditions and adoption of soil-crop management practices in different MZs. Altitude is another factor in IGP affecting soil parameters. Hence, appropriate soil management strategies need to be devised in different MZs. For example, the level of SOC content needs to be enhanced in MZ3 and MZ4 through adoption of different management practices for better soil function and nutrient availability. Though mean concentrations of available S and micronutrients in different MZs were higher than the critical range of deficiency for respective nutrients, different levels of deficiency exist in the MZs (Table 7). The MZ1 had the highest area (54.6%) with S deficiency followed by MZ5 (53.6%), MZ2 (49.8%), MZ4 (41.3%), MZ6 (35.8%), and MZ3 (32.8%). Similarly, Zn deficiency area in different MZs followed the order: MZ1 (35.8%) < MZ5 (27.5%) < MZ6 (26.7%) < MZ2 (26.5%) < MZ3 (25.3%) < MZ4 (25.1%). The MZ3 had the highest area with Fe (9.80%) and Mn (8.9%) deficiency whereas; the MZ1 had the highest area with Cu (12.3%) deficiency. The highest area under B deficiency was recorded in MZ5 (32.6%) followed by MZ1 (23.0%), MZ6 (22.7%), MZ4 (22.1%), MZ2 (21.6%) and MZ3 (11.7%). The states in IGP are having different soil problems [29] and have varied average consumption of S and micronutrients fertilizers [74] (Table 8). All the states of IGP had all 6 MZs (Table 8). Punjab had the highest % of area in MZ3 (59.4%) whereas Haryana had the largest area in MZ3 (29.3%) and MZ4 (28.1%). The state of Uttar Pradesh had higher area in MZ3 (21.8%) and MZ (21.7%). Bihar had MZ6 in 40.9% of area and West Bengal had 62.0% of area in MZ5.

**Table 6:** FPI and NCE values deciding optimum cluster numbers for the study area.

Class	FPI	NCE
2	0.866	0.897
3	0.838	0.857
4	0.799	0.805
5	0.783	0.778
6	0.747	0.729
7	0.759	0.732
8	0.766	0.735

FPI = fuzzy performance index, NCE = normalized classification entropy

**Table 7:** Mean values of soil properties and available nutrients in different management zones.

Management zone	No. of Points	pH	EC	SOC	S	Zn	Fe	Cu	Mn	B	%Area
1	9933	7.41b	0.32b	0.58b	19.9c (54.6)	0.82c (35.8)	16.6c (4.1)	1.5c (12.3)	12.4b (8.3)	0.99b (23.0)	8.8
2	7451	7.38b	0.33b	0.59a	19.0c (49.8)	0.99b (26.5)	21.5a (3.4)	2.3a (6.7)	12.6b (6.0)	0.80d (21.6)	14.1
3	9808	7.59a	0.35b	0.55c	24.1a (32.8)	1.12a (25.3)	15.2e (9.8)	1.40c (9.5)	11.2c (8.9)	1.12a (11.7)	21.7
4	6417	7.11c	0.34b	0.57c	20.2c (41.3)	1.04b (25.1)	17.9c (6.7)	1.21d (5.8)	12.8b (3.9)	0.97b (22.1)	16.2
5	9162	7.02c	0.41a	0.60a	19.3c (53.6)	1.05b (27.5)	19.3b (6.3)	1.69c (4.8)	14.1a (2.1)	0.98b (32.6)	24.4
6	12330	7.48b	0.36b	0.58b	21.7b (35.8)	1.16a (26.7)	18.2b (2.9)	1.89b (3.6)	12.1b (2.6)	0.92c (22.7)	14.8

pH = soil acidity, EC = electrical conductivity; SOC = soil organic carbon; S, Zn, Fe, Cu, Mn and B = available sulphur, zinc, iron, copper, manganese and boron in soil, respectively. The different letters in each column highlight the significant differences between the management zones at p < 0.05. Figures in parentheses indicate % area of deficiency of individual nutrient in each management zone.

**Table 8:** States in IGP with different predominant soil problems, average consumption of S and micronutrients fertilizer and MZs.

States in IGP	Predominant soil problem	Average consumption of S and micronutrients fertilizers during 2012–13 to 2016–17	Soil MZs	% Area
Punjab	Alkalinity	9958 metric ton S containing fertilizers, 29324 metric ton zinc sulphate, 40 metric ton borax/boric acid, 2097 metric ton ferrous sulphate, 1365 metric ton manganese sulphate and 37 metric ton copper sulphate	MZ1	2.80
			MZ2	11.0
			MZ3	59.4
			MZ4	12.7
			MZ5	9.00
			MZ6	5.10
Haryana	Sodicity and alkalinity	12766 metric ton S containing fertilizers, 21446 metric ton zinc sulphate, 0 metric ton borax/boric acid, 0 metric ton ferrous sulphate, 0 metric ton manganese sulphate and 0 metric ton copper sulphate	MZ1	1.70
			MZ2	4.40
			MZ3	29.3
			MZ4	16.9
			MZ5	19.7
			MZ6	28.1
Uttar Pradesh	Alkalinity and sodicity	65614 metric ton S containing fertilizers, 23957 metric ton zinc sulphate, 0 metric ton borax/boric acid, 0 metric ton ferrous sulphate, 0 metric ton manganese sulphate and 0 metric ton copper sulphate	MZ1	16.8
			MZ2	14.9
			MZ3	21.8
			MZ4	17.2
			MZ5	21.7
			MZ6	7.70
Bihar	Alkalinity	32622 metric ton S containing fertilizers, 1352 metric ton zinc sulphate, 101 metric ton borax/boric acid, 0 metric ton ferrous sulphate, 0 metric ton manganese sulphate and 0 metric ton copper sulphate	MZ1	0.10
			MZ2	24.2
			MZ3	11.3
			MZ4	10.8
			MZ5	12.8
			MZ6	40.9
West Bengal	Acidity	52276 metric ton S containing fertilizers, 9734 metric ton zinc sulphate, 7333 metric ton borax/boric acid, 260 metric ton ferrous sulphate, 500 metric ton manganese sulphate and 484 metric ton copper sulphate	MZ1	4.30
			MZ2	6.40
			MZ3	3.90
			MZ4	22.0
			MZ5	62.0
			MZ6	1.40

Based on this information nutrient management decisions and supply of nutrients to different zones could be prioritized (Table 9). The MZs having higher deficiency level of a particular nutrient need to receive first attention followed by other MZs having subsequently low levels of deficiency. The MZ3 needs to be paid more attention compared to other MZs for Fe and Mn management. Similarly, B management needs to be prioritized in MZ5, MZ1 and MZ6 compared to other MZs. This will help in optimum utilization of resources. Considering the available S and micronutrient status in soils and per cent area of deficiency, different quantities of customized multi-nutrient mixture fertilizers in different grades could be provided to different MZs for efficient nutrient management. In addition, the farmers and farm mangers could suitable be advised to grow efficient and inefficient crop cultivars in different MZs based on soil nutrient status and resource availability. Nutrient-efficient and nutrient-inefficient cultivars of the crops behave differently (in terms of crop growth and yield) under different soil nutrient status and management practices. For example, resource poor farmers could grow nutrient efficient crop cultivars in nutrient deficient soil and could obtain good crop yield without application of that nutrient and vice versa. It is, therefore, pertinent for the farmers and farm managers of IGP to take cognizance of soil parameters in different MZs to devise simple, easy, cost efficient soil-crop manipulation ways for higher and sustainable crop production.

**Table 9:** Nutrient management priority for different management zones.

Nutrient	Management priority
S	MZ1 > MZ5 > MZ2 > MZ4 > MZ6 > MZ3
Zn	MZ1 > MZ5 > MZ6 > MZ2 > MZ3 > MZ4
Fe	MZ3 > MZ4 > MZ5 > MZ1 >MZ2 > MZ6
Cu	MZ1 > MZ3 > MZ2 > MZ4 >MZ5 > MZ6
Mn	MZ3 > MZ1 > MZ2 > MZ4 >MZ6 > MZ5
B	MZ5 > MZ1 > MZ6 > MZ4 >MZ2 > MZ3

## Conclusion

The study revealed wide spatial variability with moderate (except strong for SOC) spatial dependence for phyto-available S, Zn, B, Fe, Mn and Cu and associated soil properties (pH, EC and SOC) in IGP, India. The concentration of S and micronutrients variedly widely with deficiency level of 67.7% for S, 46.9% for Zn, 44.7% for B, 137% for Fe, 18.3% for Mn and 15.3% for Cu. The range values of the semi-variograms of different soil parameters could be considered for designing future soil sampling strategies in IGP of India. Principal component analysis and fuzzy c*-*means clustering resulted in 6 MZs of IGP having significantly different values of soil parameters. The generated MZ maps could be used for zone-specific manipulation of available S and micronutrients for sustainable crop production. Further, this study also revealed that soil MZs could be delineated in other cultivated regions of the world for zone specific nutrient supply and management.

## Supporting information

S1 Data (RAR)

S2 Data (RAR)
